# The effects of ten weeks resistance training on sticking region in chest-press exercises

**DOI:** 10.1371/journal.pone.0235555

**Published:** 2020-07-09

**Authors:** Atle Hole Saeterbakken, Vidar Andersen, Roland van den Tillaar, Florian Joly, Nicolay Stien, Helene Pedersen, Matthew Peter Shaw, Tom Erik Jorung Solstad

**Affiliations:** 1 Faculty of Education, Arts and Sports, Western Norway University of Applied Sciences, Sogndal, Norway; 2 Department of Sport Science and Physical Education, Nord University, Levanger, Norway; 3 Rennes School of Sports, Rennes, France; University of Belgrade, SERBIA

## Abstract

The aim of the study was to compare the effects of a 10-week chest-press resistance training on lifting regions in a trained exercise and a none-trained exercise; the barbell bench press (BBP). Thirty-five resistance trained men with 4.2 (± 2.3) years of resistance training experience were recruited. The participants were randomized to attend a resistance program, performing the chest-press, twice per week using either, Smith machine, dumbbells or laying on Swiss ball using a barbell. A six-repetitions maximum (6RM) test was conducted pre- and post-training in the trained chest-press exercise and non-trained BBP to examine lifting velocity, load displacement and the time of the pre-sticking, sticking and post-sticking regions. Additionally, the muscle activity in pectoralis major, triceps brachii, biceps brachii and deltoid anterior was examined. In the post-test, all three chest-press groups decreased lifting velocity and increased the time to reach the sticking- and post-sticking region. Independent of the type of chest-press exercise trained, no differences were observed in vertical displacement or in the muscle activity for the three lifting regions. In general, similar changes in kinematics in trained exercise and those observed in the BBP were observed for all three groups. This indicates that none of the three chest-press exercises (Swiss ball, Smith machine or dumbbells) were specific regarding the lifting regions but displaced a transferability towards the non-trained BBP. However, improved strength altered the sticking region among resistance trained men.

## Introduction

Barbell bench press (BBP) is one of the most frequently used exercises to improve strength in the upper body. BBP performance is measured as the maximum load an athlete can lower to the chest and pressing up until the elbows are fully extended. In addition to be an independent competition, BPP is one of three exercises in Powerlifting. However, bench press practitioners use several chest-press exercises in their training routines. The most frequently used chest-press exercises include dumbbell and training machines. Lately, unstable surfaces have also been used as supplemental exercises to BBP. BBP performance has been examined in several studies [1–3] and the cause of failure has gained increased interest in the last decades [4–6].

In BBP, a lifting region where subjects can produce less force (i.e. resulting in reduced barbell velocity) compared to other regions has been identified and called the stick region [5, 7–9]. The sticking region appears during maximal or close to maximal loads lifted in the upwards movement [5, 6]. Still, little is known about what causes this reduction in force generation or whether resistance training may change the BBP kinematic. For example, Elliott et al. [7] demonstrated no reduced neural drive for the prime movers in the sticking region. Furthermore, Tillaar and Ettema [5] showed a sequence peak activation pattern in the sticking region, whereas pectoralis major and deltoid anterior reached their peak activation around the sticking point (the lowest upward velocity of the barbell) [5]. Nonetheless, the majority of the current findings have demonstrated similar muscle activity in the prime movers when comparing the lifting regions [4, 5, 10]. To the authors knowledge, no previous studies have examined muscle activity and lifting regions in BBP after a training intervention, but one study has examined lifting velocity in 1RM after a six-week intervention [11]. The authors demonstrated a 9.3% improvement in 1RM strength in BBP, but only a 0.02m/s change in lifting velocity which was independent of relative strength [11].

Several studies have suggested that a poor mechanical force position of the prime movers in the sticking region reduces the capacity to generate force [6–8]. However, when comparing successful and unsuccessful attempts in bench press, only half of the failures occur in the sticking region [4]. Furthermore, Elliott et al. [7] demonstrated no increase in the moment arm of the shoulder and elbow in the sticking region. Decreased diminishing potentiation caused by the elastic components has also been used to explain the sticking region. For example, Tillaar and colleagues [9] examined maximal isometric contraction (no diminishing potential) in 12 different positions in BBP. They demonstrated lower force output in the sticking region associated with similar electromyographic (EMG) activity of the prime movers [9]. Similar reduced barbell velocity has also been observed comparing BBP were the ascending phase started immediately after the descending phase (diminishing potential) compared to only performing the ascending phase (no diminishing potential) [12]. Both findings support the speculation that poor mechanical force position is the cause of sticking region. The sticking region has also been demonstrated in chest-press exercises with different stability requirements (e.g. Smith-machine, BBP and dumbbells) [10]. Different stability requirements (i.e. Smith-machine < barbell < dumbbells) resulted in longer sticking regions, but similar activation in pectoralis major.

Despite a growing interest in the sticking region, little is known about the changes in the sticking region after a period of resistance-training or if these possible changes in kinematics are specific to the trained chest-press exercise or are transferred to other chest press exercises (i.e. BBP). However, several studies have demonstrated improved upper-body strength and power after performing bench press training using only a limited ROM [13–15]. None of these studies examined sticking region in chest-press but instead they examined power output, isometric force and peak force after training bench press with different range of motion. However, training in a limited range of motion (ROM) that demands maximal force production (often referred to as the accentuation principle) has proven favorable to generate power and force in these ROMs compared to training full ROM in BBP [13]. The maximal demands of force production in BBP is beyond the sticking region in BBP [5, 9, 12] and it could be an argument to use the post-sticking region to generate maximal force in BBP. Consequently, knowledge of the sticking region after a training period, but also possible changes in kinematics in trained and none-trained chest-press exercise, may help coaches and athletes to design better chest-press programs.

Furthermore, using different chest-press exercises with different stability requirements, may stress the neuromuscular system differently. It has previously been speculated that instability in resistance training can favor force production in more stable exercises [16, 17]. For example will chest press with dumbbells on bench represent unstable loads performed on a stable surface, while barbell press laying on a Swiss ball will represent an unstable surface with a relatively stable load [18]. In contrast, bench press in a Smith machine represent a stable surface and stable load which may favor force production [19, 20]. Therefore, and to the authors’ best knowledge, no previous studies have examined the possible changes in sticking region after attending a training intervention in chest-press exercises. The aim of the present study was two-folded: 1) to examine possible changes in the sticking regions after a 10-week chest-press training using either a Smith machine, dumbbells or barbell chest-press on a Swiss ball and 2) to examine whether these possible changes in the kinematics in trained exercise were transferred to the non-trained exercise barbell bench press. We hypothesized changes in lower barbell velocity and longer lasting sticking region in trained chest-press sticking, but similar kinematics in the non-trained barbell bench press.

## Methods

### Design

To compare the effects of ten weeks training of different chest-press exercises on the sticking region in bench press, all participants were randomized to one of three chest press groups: chest press using a Smith Machine, chest-press with dumbbells and barbell chest-press on a Swiss ball. The participants trained their selected exercise twice per week over a 10-weeks period. Pre and post intervention, the participants were tested in six repetitions maximum (6RM), electromyographic activity of the prime movers (pectoralis major, deltoid anterior, triceps brachii and biceps brachii) and kinematics (velocity, load displacement and the time of the occurring of the pre-sticking, sticking and post-sticking regions) in the trained chest-press exercises and the traditional BBP. All participants trained BBP weekly before the intervention. However, during the intervention, none of the participants trained the BBP which was defined as a neutral exercise.

### Participants

Thirty-five resistance-trained men with a minimum of two years of resistance training experience were recruited using social media, personal communication, fliers and presentations of the project among students at campus Sogndal, Norway. To be included, the participants had to be men, free of pain that may have prevented maximal effort, be able to lift at least their own body weight in 1RM in BBP and train BBP weekly in their regular program six months prior to the start of the intervention. Furthermore, the participants had to perform the four chest-press exercises with consistence and good technique as described later. If any of the participants had pain which prevented them to maximal effort, were not able to lift their body weight in 1RM in BBP or did not train BBP weekly the last six months, they were excluded to participate. None of the participants were competitive powerlifters or weightlifters. The participants (age: 22.66 ± 2.06 years, height: 1.80 ± 0.06 m, weight: 78.62 ± 7.05 kg, Table 1) had 4.15 ± 2.24 years of resistance training experience with a relative 6RM strength (6RM loads / body weight) of 0.97 in BBP.

**Table 1:** The subjects’ characteristics.

Training group	Height (m)	Age (years)	Body mass (kg)	6RM strength bench presses (kg)
Smith machine (n = 12)	1.82 ± 0.06	22.4 ± 1.6	78.6 ± 6.5	72.7 ± 9.5
Swiss ball (n = 12)	1.79 ± 0.06	23.3 ± 2.1	78.4 ± 7.0	80.4 ± 19.9
Dumbbells (n = 11)	1.80 ± 0.06	22.2 ± 2.6	78.6 ± 8.0	76.7 ± 10.6

### Ethics statement

All participants were informed orally and in writing of the study procedures and the possible risks. Informed written consent was obtained from the participants before inclusion in the study. The study was approved by the Norwegian Centre of Research data and conformed to the Helsinki Declaration (2013). The study was carried out in accordance with the guidelines in the Declaration of Helsinki, national and international laws and regulation [21].

### Procedures

#### Testing

**Fig 1:**
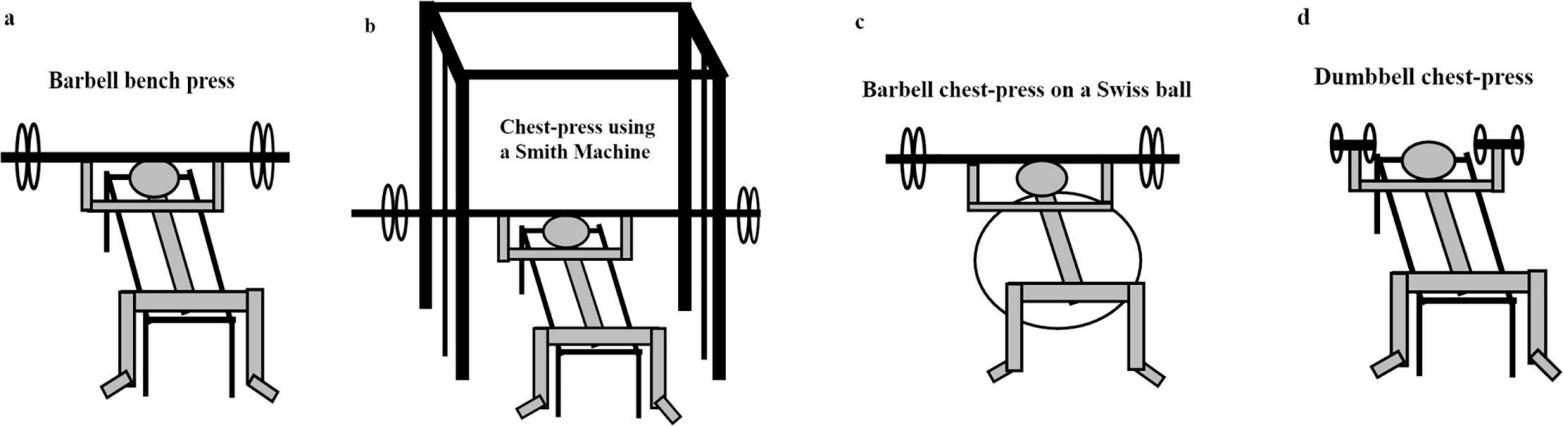
The four chest press exercises: a; traditional barbell bench press, b; chest press using a Smith machine, c; chest press on a Swiss ball and d; dumbbell chest press.

After two familiarization sessions (separated by 2–5 days), 6RM in all four chest exercises (traditional barbell bench press, barbell chest press on a Swiss ball, dumbbell chest press and chest press using a Smith machine; Fig 1) was determined (pre-test). When changing exercises, 2–3 repetitions with non-exhausting loads (approximately 50% of 6RM loads) were conducted to familiarize to new exercise. The order was randomized and counterbalanced, but was identical in the pre-and post-test. All testing procedures in the exercises have been described in previous studies [3, 10, 22]. The barbell (free weight, Swiss ball and Smith machine exercises) had to be lowered and lightly touch the chest before being lifted until the elbows were extended. When testing dumbbell exercise, an elastic band was placed across the dumbbells, which had to touch the chest. The grip width using the barbell was self-selected, but identical in all three barbell exercises (measured and controlled before each exercise and testing time). When using the bench (dumbbells, free weight and Smith machine exercises) the head, shoulder and hips had to be in contact with the bench during lifting. In the Swiss ball exercise, legs were elevated to match the height of a traditional bench based on an 80kg person lying on the Swiss ball and lifting 80 kg (i.e. ball pressure of 3.0 psi). The foot width was self-selected, but identical and controlled before each testing session and exercise.

The warm-up consisted of 20, 10 and 8 repetitions using 20%, 50% and 70% of their self- estimated 6RM in traditional bench press respectively. After the first session, the participants`achieved 6RM loads were used to determine the warm-up load in the pre-test. In the familiarization sessions and before pre- and post-tests, the traditional barbell bench press was used during the warm-up.

#### Training

After the pre-test, the participants were randomized, by drawing, to one of the three training groups (Swiss ball, dumbbells or Smith machine). The allocated exercise was used in the warm-up procedures during the training sessions. All participants trained twice per week over a 10-weeks period. Each session was separated by at least 48 hours. During the intervention period, the participants were not allowed to train exercises targeting the prime movers in bench press (i.e. pectoralis, triceps and deltoids). The training load was approximately 85% of 1RM and was performed as six repetitions for four sets. If the participant was able to perform the six repetitions in the fourth set, the load was increased at the next session by 2.5–5.0 kg. Two test-leaders monitored testing and training, encouraged the participants and ensured correct execution of the exercises.

#### Measurements

To examine the kinematics (time, velocity and vertical displacement) during the 6RM lifts, a linear encoder ET-Enc-02 (Ergotest Innovation AS, Porsgrunn, Norway) was used. The encoder was attached to the barbell or dumbbell and placed below the loads. The time was calculated from the turnover from then descending phase / ascending phase to the pre-sticking, sticking and post-sticking regions. The encoder had a resolution of 0.019 mm and a sampling rate of 200Hz [23]. Typically, the final repetition is too close to fatigue and differs too much from the other repetitions [6, 24]. Therefore, only the upward movement of the fifth repetition was used in the analyses.

**Fig 2:**
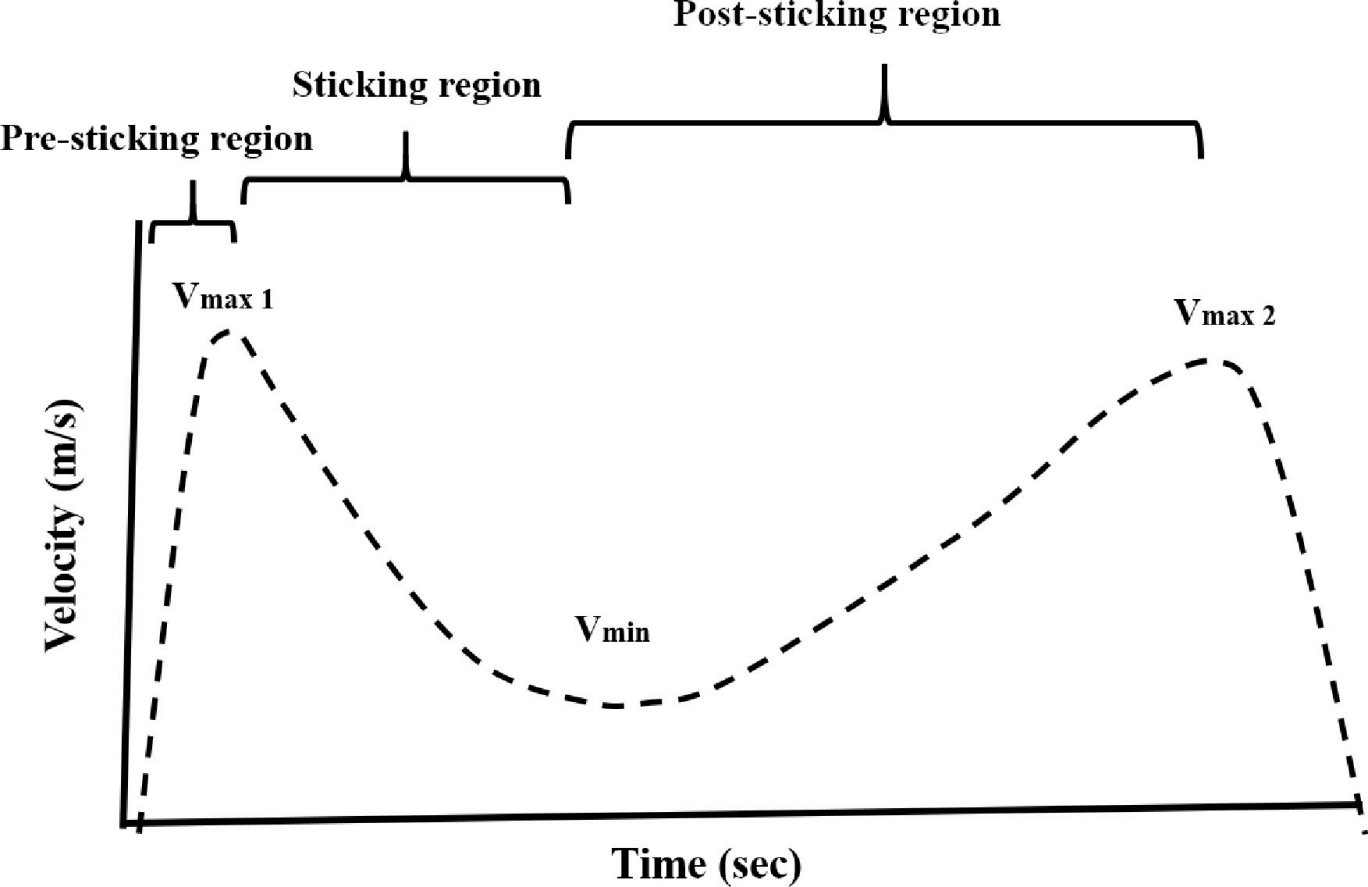
A typical example of the sticking region in barbell bench press.

Using the Musclelab software V8.10 (Ergotest Innovation AS, Porsgrunn, Norway), the pre-sticking, sticking and post-sticking region were identified [9, 24, 25] (see Fig 2). The pre-sticking region was identified as the lowest barbell position (turnover from descending to ascending phase) until the first peak velocity (V_max1_). The sticking region was identified as the region between first peak velocity (V_max1_) and lowest lifting velocity (V_min_). Last, the post-sticking region was region between the lowest lifting velocity (V_min_) and the second peak velocity (V_max2_). For each of the three regions, the barbell velocity, vertical displacement of the barbell and the time from the lowest barbell position (i.e. the turning point from the descending to the ascending phase) were identified and used in the analyses.

A commercial EMG recording system (Musclelab 4020e, Ergotest Innovation AS, Porsgrunn, Norway) was synchronized with a linear encoder to measure the mean muscle activity of the pectoralis major, deltoid anterior, biceps brachii and triceps brachii of the three regions in BBP. Before placing the self-adhesive electrodes (Dri-stick silver Circular sEMG electrodes AE-131, NeuroDyne Medical, Cambridge, MA, USA), the skin was shaved, abraded and washed with alcohol according to previous studies and recommendations [26]. The electrodes had an 11 mm contact diameter with a 20 mm center-to-center distance and were placed in the direction of the muscle fiber using anatomic landmarks, according to the recommendations of SENIAM and previous studies [22, 26, 27]. The EMG signal was amplified and filtered using a preamplifier. The preamplifier had a common mode rejection rate of 100 db. The raw EMG signal was then band-pass filtered (fourth-order Butterworth filter) with a cutoff frequency of 8–600 Hz. The EMG signal was converted to root-mean-square (RMS) using a hardware circuit network with an average constant of 100 ms (total error ± 0.5%) and a frequency response 0–600 kHz. Finally, the RMS-converted signal was re-sampled at a rate of 100 Hz using a 16-bit analog-to-digital converter (AD637). The RMS-values were normalized using an isometric barbell bench press with a 90° angle in the shoulder and elbow joints [22]. The participants were instructed to perform two maximal voluntary contractions (MVC) lasting five seconds whereby the attempt with the greatest RMS values (over a period of three seconds) was used. Each MVC was separated by 1–2 minutes.

#### Statistics

All data were examined using SPSS (version 25.0; SPSS, Inc., Chicago, IL, USA). To examine the kinematics (time, vertical displacement and velocity) and EMG activity (pectoralis major, deltoid anterior, biceps brachii and triceps brachii) in the trained chest-press exercises and BBP after a 10-week chest-training program, a two-way repeated measure analyses of variance (ANOVA) [exercise (training exercise, BBP)] x [time (pre, post)] was used in each of the three lifting regions (pre, sticking and post-sticking region) for each training group. If interaction or main effects were detected, Bonferroni post-hoc correction was used to identify where the differences were. Comparisons between the three regions or between the three training groups were not analyzed as the differences are well explored [5, 9, 10, 25] and the aim of this study was to examine possible specific changes in the sticking region in trained exercise and the transferability to BBP after the chest-press training. To examine possible base line differences in anthropometric data, resistance training experience or 6RM strength between the groups, a one-way ANOVA with Bonferroni post-hoc correction was used. Dependent t-tests were used to examine 6RM improvement in trained chest press exercise and BBP. Statistical significance was accepted at p < 0.05. All results are presented as mean ± SD.

## Results

At pre-test, there were no significant differences in anthropometric, resistance training experience or 6RM strength between the groups (F = 0.684–1.118, eta = 0.653–0.807 and p = 0.220–1.000). All the 6RM loads increased in the trained exercises (i.e. Smith machine, Swiss ball and dumbbell) as well as BBP. All details are presented in Table 2.

**Table 2:** The 6RM improvements in the chest-press exercises.

Training group	Exercise	Pre-test (kg)	Post-test (kg)	Improvement (%)	p-values
Smith machine	Smith machine	68.8 ± 8.7	84.0 ± 8.8	22.7 ± 8.3	< 0.01
	Barbell bench press	71.0 ± 9.5	82.3 ± 7.0	17.0 ± 11.3	< 0.01
Swiss ball	Swiss ball	73.1 ± 18.1	95.6 ± 20.9	32.5 ± 15.8	< 0.01
	Barbell bench press	78.3 ± 20.3	86.3 ± 18.3	11.5 ± 7.2	< 0.01
Dumbbells	Dumbbell	60.8 ± 9.8	82.7 ± 6.0	38.6 ± 19.5	< 0.01
	Barbell bench press	75.3 ± 10.9	83.7 ± 11.0	11.6 ± 7.0	< 0.01

### Kinematics

#### Smith machine group

**Fig 3:**
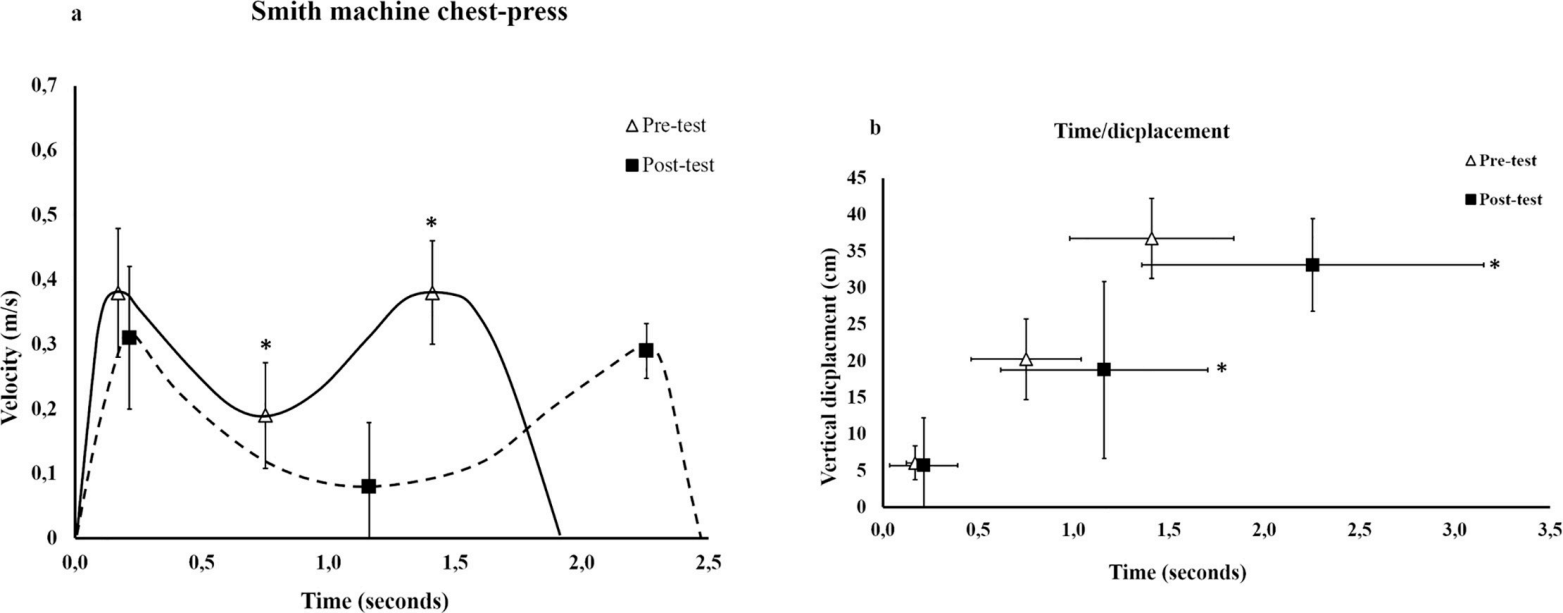
The mean changes ± standard deviation in barbell velocity (a), lifting time and barbell displacement (b) pre-and post-training in the Smith machine group in trained exercise. * significant different than pre-test (p < 0.05).

For the Smith machine group, there was no interaction between testing time (pre, post) and exercise (trained Smith machine, BBP) (F = 0.006–3.5, p = 0.077–0.930) in any of the regions for the variables the barbell`s vertical displacement, lifting velocity and the occurring (time) of the different regions in the ascending phase. No main effect for exercise (F = 0.14–2.7, p = 0.110–0.720) was observed in any of regions, but a main effect in testing time was observed for the variables velocity and time (F = 5.3–29.4, p < 0.001), but not for the vertical displacement (F = 2.5–3.6, p = 0.070–0.125) in the three regions. All post hoc tests of the changes pre- and post-testing and between trained exercise (Smith machine) and BBP are presented in Table 3, Fig 3A and 3B and S1 Table.

**Table 3:** The kinematics (mean ± standard deviation) pre-and post-training in barbell bench press.

Lifting phase	Training group		Vertical displacement (cm)	Time (sec)	Velocity (m/s)
Pre- sticking region	SM#	Pre	7.92 ± 2.76	0.25 ± 0.15	0.32 ± 0.07
	SM	Post	6.05 ± 2.67	0.17 ± 0.06	0.28 ± 0.08*
	SW#	Pre	6.55 ± 2.08	0.18 ± 0.04	0.33 ± 0.08
	SW	Post	6.19 ± 1.92	0.22 ± 0.09	0.28 ± 0.07*
	DB#	Pre	6.90 ± 4.74	0.25 ± 0.18	0.28 ± 0.13
	DB	Post	5.94 ± 4.08	0.26 ± 0.20	0.26 ± 0.09
Sticking region	SM	Pre	18.27 ± 5.43	0.67 ± 0.19	0.21 ± 0.08
	SM	Post	14.96 ± 6.11	0.94 ± 0.37	0.04 ± 0.11*
	SW	Pre	19.29 ± 5.99	0.77 ± 0.35	0.17 ± 0.08
	SW	Post	18.18 ± 7.98	0.84 ± 0.48	0.08 ± 0.08*
	DB	Pre	17.00 ± 9.88	0.69 ± 0.37	0.16 ± 0.06
	DB	Post	17.41 ± 7.96	1.03 ± 0.40*	0.09 ± 0.09
Post- sticking region	SM	Pre	35.79 ± 3.73	1.31 ± 0.24	0.39 ± 0.08
	SM	Post	32.45 ± 6.98	2.11 ± 0.85*	0.33 ± 0.09*
	SW	Pre	35.67 ± 5.52	1.48 ± 0.37	0.35 ± 0.09
	SW	Post	34.11 ± 6.52	2.15 ± 0.59*	0.27 ± 0.09
	DB	Pre	34.36 ± 6.27	1.39 ± 0.27	0.37 ± 0.10
	DB	Post	32.71 ± 5.18	2.10 ± 1.06	0.33 ± 0.07**

*significant differences between pre- and post training (p < 0.05).

** significant differences between trained exercise and barbell bench press.

#SM = Smith machine, SW = Swiss ball and DB = Dumbbell

#### Swiss ball group

**Fig 4:**
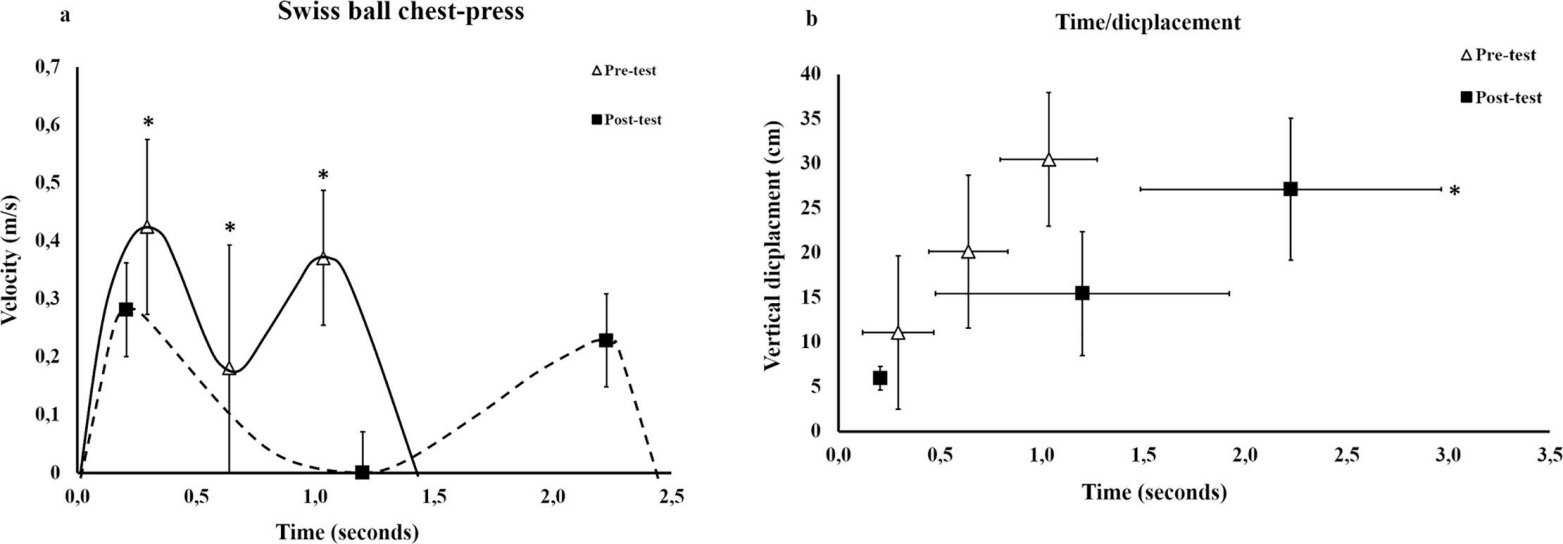
The mean changes ± standard deviation in barbell velocity (a), lifting time and barbell displacement (b) pre-and post-training in the Swiss ball group in trained exercise. * significant different than pre-test (p < 0.05).

For the Swiss ball group, no interactions between testing time (pre, post) and exercise (trained Swiss ball, BBP) were observed in any of the three regions (F = 0.098–4.3, p = 0.051–0.761) for the variables the barbell`s vertical displacement, lifting velocity and the occurring (time) of the different regions in the ascending phase. Furthermore, there was no main effect in exercise (F = 0.001–2.6, p = 0.125–0.982), but a main effect for testing time was observed (F = 9.1–21.7, p < 0.001–0.007). All post hoc tests of the changes pre- and post-tests in trained exercise (Swiss ball) and BBP are presented in Table 3, Fig 4A and 4B and S1 Table.

#### Dumbbell group

**Fig 5:**
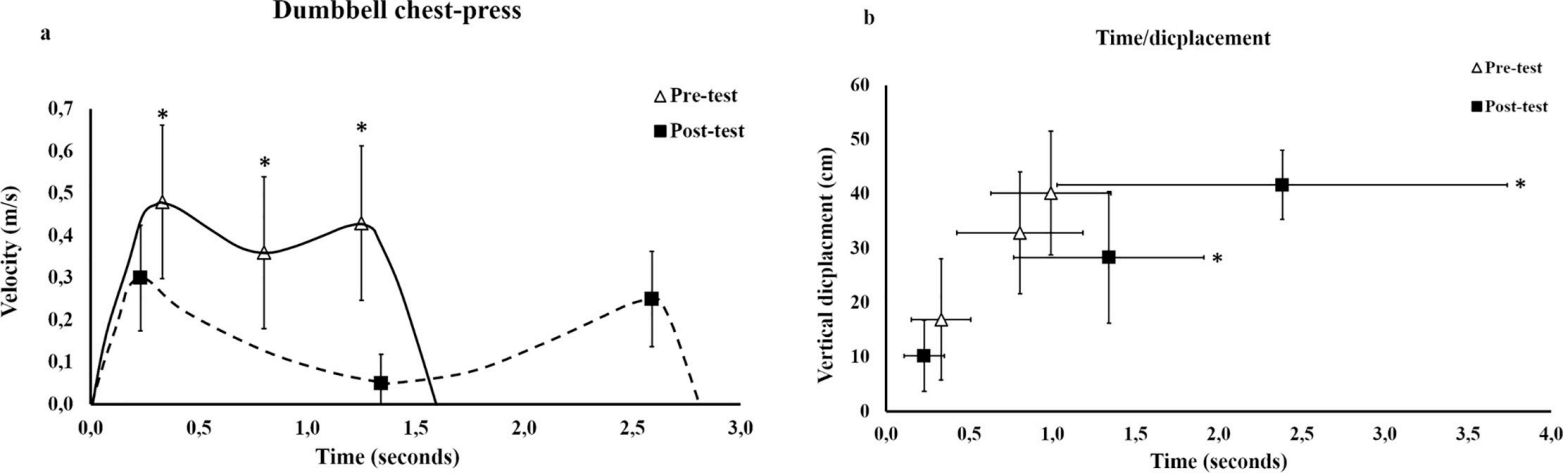
The mean changes ± standard deviation in barbell velocity (a), lifting time and barbell displacement (b) pre-and post-training in the Dumbbell group in trained exercise. * significant different than pre-test (p < 0.05).

For the dumbbell group, an interaction between testing time (pre, post) and exercise (trained dumbbell exercise, BBP) was observed for the dumbbell`s velocity in all three regions (F = 4.6–13.5, p = 0.002–0.044), but not for vertical displacement (F = 0.27–2.3, p = 0.153–0.614) or time (F = 0.69–3.43, p = 0.079–0.425. However, for the variable vertical displacement of the dumbbells and the occurring of the three regions (time), main effects for testing time (F = 14.6–23.0, p <0.001–0.001) and exercise (F = 5.4–11.9, p = 0.003–0.034) were observed. All post hoc tests for the trained exercise (dumbbell) and BBP of the changes pre- and post-testing are presented in Table 3, Fig 5A and 5B and S1 Table.

### Muscle activation (EMG)

No interaction between exercise (trained exercise, BBP) and time (pre, post) for the EMG activity in triceps brachii, biceps brachii, deltoid anterior and pectoralis major in any of the three lifting regions was observed for any of the three training groups (F = 0.001–3.9, p = 0.063–0.98) with exception of the triceps brachii activation in the post-sticking region for the dumbbell group (F = 7.5, p = 0013). Post hoc tests demonstrated greater triceps brachii activity in the pre-test than post-test in trained dumbbell chest-press exercise (p = 0.008), but not BBP (p = 0.75). No differences were observed in triceps brachii between trained exercise and BBP in the pre-test (0.27) or post-test (p = 0.530).

The main effects for time (pre, post) for the four muscles in the three lifting regions were F = 0.01–10.8, p = 0.004–0.94. Post hoc tests only demonstrated greater triceps brachii activity in the pre-test than the post-test in trained exercise (p = 0.008) for the dumbbell group in the post-sticking region. For the other muscles, no differences were observed in any of the three regions for any of the training groups (p = 0.273–0.530). Percentage changes in muscles activity between pre-and post-tests are presented in Table 4.

**Table 4:** Percent changes in normalized EMG activity in trained chest-press exercise and barbell bench press. All values are presented as mean ± standard derivation.

Lifting phase	Training group	Exercise	Pectoralis major	Triceps brachii	Deltoid anterior	Biceps brachii
Pre-sticking region	SM¤	Trained	7.9 ± 69.2	5.2 ± 47.2	-4.2 ± 32.0	2.2 ± 69.2
	SM	BBP	14.1 ± 55.3	11.7 ± 39.0	8.3 ± 38.6	25.7 ± 106.7
	SW¤	Trained	10.6 ± 43.2	-13.1 ± 65.1	-8.5 ± 22.1	14.1 ± 55.3
	SW	BBP	0.6 ± 80.5	-9,0 ± 27.1	2.2 ± 35.1	5.4 ± 93.5
	DB¤	Trained	-11.9 ± 38.6	-33.6 ± 38.5 #	-28.1 ± 27.1	17.3 ± 88.9
	DB	BBP	1.6 ± 45.0	-18.0 ± 29.4 **	0.3 ± 0.1	15.3 ± 76.4
Sticking region	SM	Trained	7.3 ± 68.4	12.2 ± 69.7	0.2 ± 37.0	15.1 ± 76.0
	SM	BBP	9.2 ± 59.9	47.3 ± 101.4	-2.3 ± 37.2	5.4 ± 91.0
	SW	Trained	10.6 ± 43.1	-25.6 ± 42.1	-8.6 ± 22.1	4.0 ± 55.7
	SW	BBP	18.5 ± 69.7	-10.0 ± 30.1	-8.2 ± 26.0	30.7 ± 88.3
	DB	Trained	-18.5 ± 28.1	-43.1 ± 27.2	-28.1 ± 27.23	-21.1 ± 48.5
	DB	BBP	17.4 ± 8.0	9.0 ± 63.4	25.6 ± 26.5	1.4 ± 45.6
Post-sticking region	SM	Trained	-19.90± 35.1	-0.1 ± 37.9	-1.4 ± 45.8	26.1 ± 89.2
	SM	BBP	0.6 ± 41.1	15.4 ± 49.4	-5.2 ± 25.7	26.1 ± 94.1
	SW	Trained	-5.0 ± 51.3	1.5 ± 0.4	-17.5 ± 39.5	32.7 ± 94.1
	SW	BBP	1.7 ± 44.5	3.6 ± 39.0	-9.5 ± 34.2	35.0 ± 97.4
	DB	Trained	-30.7 ± 20.6	-47.2 ± 31.9 *	-24.7 ± 35.2	-20.5 ± 44.1
	DB	BBP	-0.1 ± 23.5	2.1 ± 1.1	28.4 ± 25.5	-11.4 ± 41.9

*significant differences between pre- and post-tests (p < 0.05).

# significant differences between trained exercise and barbell bench press at pre-testing (p < 0.05).

** significant differences between trained exercise and barbell bench press at post-testing (p < 0.05). ¤SM = Smith machine, SW = Swiss ball, DB = Dumbbell and BBP = barbell bench press.

The main effects for exercise (trained, BBP) for the four muscles in the three lifting regions were F = 0.006–10.9, p = 0.004–0.944. Post hoc tests only demonstrated greater triceps brachii activation in BBP than dumbbell chest-press for the dumbbell group in both pre- and post-test in the pre-sticking region (p = 0.034 and p = 0.030). For the other muscles, no differences were observed in any of the three regions for any of the training groups (p = 0.068–1.000). Percentage changes in muscles activity between pre-and post-tests are presented in Table 4.

## Discussion

The main finding of the present study was that the sticking region in trained chest-press exercises changed after a 10-week training period. All three training groups decreased lifting velocity but increased the duration (i.e. time) before the sticking region. Furthermore, when examining the three lifting regions, there were no differences in vertical displacement between pre- and post-training independent of trained exercise. In the post-test, no differences were observed in the muscle activity or comparing kinematics in the trained exercise and BBP. Instead, similar changes in sticking region in the trained exercises and those observed in the BBP were shown for all three groups. This indicates that none of the three chest-press exercises (Smith machine, dumbbells or barbell chest-press on a Swiss ball) were specific regarding lifting kinematics but produced a transferability towards the non-trained barbell bench press.

All groups improved their 6RM in trained exercise by ~23–39%. Still, all groups demonstrated decreased lifting velocity in the pre-sticking region with exception of the Smith machine group. However, the Smith machine group had the lowest stability requirements [3] which may allow for maximal force generation in the ascending phase instead of prioritizing stabilizing the joints, which might occurring using dumbbells or laying on a Swiss ball [28, 29]. Identical intensities (6RM) were used pre-and post and in according with the hypotheses, the velocity in the pre-sticking region decreased. It could be speculated that the participants were more tolerant of lifting higher loads but were not able to improve the acceleration (F = m x a). which explain the lower lifting velocity. Furthermore, the training program in the present study was not designed to improve explosive characteristics, but aimed to improve maximal strength. Greater 6RM load does not necessarily alter the ability to develop force rapidly.

Independent of training groups, no differences were observed in vertical displacement or time in the pre-sticking region between pre- and post-testing. This means that the pre-sticking time and barbell displacement seems independent of the increase in strength or lower pre-sticking velocity. Even so, lower lifting velocity indicates a lower acceleration in the pre-sticking phase which supported the hypotheses that improved 6RM strength improves the tolerance of lifting greater loads, but not the acceleration in the pre-sticking region.

In the sticking region, the lifting velocity decreased for all three training groups as hypothesized. Lower lifting velocity was most likely a result of lower pre-sticking velocity observed in the post-test. Furthermore, the participants used longer time to reach the sticking region, but there was no difference in the vertical displacement. This support previous findings that poor mechanical force position is the most likely explanation of the sticking region [5, 7–9, 30] as all three groups demonstrated a sticking region in their allocated chest-press exercise and BBP. Furthermore, and according to previous studies [4, 7, 9, 31], the participants demonstrated a sticking-region while lifting 6RM loads independent of exercise. However, the present study is the first to demonstrate a sticking region after improving 6RM strength independent of chest-press exercise.

In the post-sticking region, similar findings were observed across the three training groups; decreased lifting velocity, increased time spent reaching the post-sticking region, and no difference in the vertical displacement after the training intervention. These results are most likely caused by the changes observed in the pre- and sticking region. However, all training groups demonstrated an increase in velocity of the loads in the post-sticking region where the maximal force production has been demonstrated [5, 9]. In other words, chest-press enthusiasts training with ¼—¾ of the full range of motion [13], can use the three chest-press exercises to maximize force production. Importantly, the occurring of the post-sticking region varied between the exercises (~27–42 cm, S1 File) which coaches and athletes needs to remember training the respectively chest-press exercises using limited ROM.

To the authors’ knowledge, this is the first study to examine the changes in chest-press sticking region after a 10-week resistance training intervention. The present findings are difficult to compare to previous studies as only acute studies have been conducted trying to explain the occurring of the sticking region in traditional bench press [5, 7–9, 30]. Still, one study has demonstrated different lengths of the lifting regions between barbell bench press, chest press with dumbbells and in a Smith machine [10]. Irrespective of this, the aim of the present study was not to compare changes between training groups, but to examine the changes in the sticking region after training a chest-press training period.

As expected, and according to previous studies [13, 22], the present study demonstrated similar EMG activity pre- and post-training in all regions in both the trained chest-press exercise and barbell bench press. The only difference observed was greater triceps brachii activation (both pre and post) in the pre-sticking region using BBP compared to the dumbbell chest-press exercise. Lower triceps brachii activation using dumbbells is most like a result of lifting two independent loads which have been proven previously [3, 10]. In comparisons, beginners in resistance training typically increased neural drive [32, 33] and the ability to synchronize the agonist-antagonist activation [34]. In the present study, similar muscle activity in the pre-and post-tests may reflect the performance level of the participants as resistance trained, but also that the three chest-press exercises stimulate the prime movers almost identical. Previous studies have demonstrated similar muscle activity between the lifting regions [4, 5, 10]. Nonetheless, these findings were limited by only comparing different regions acutely (i.e. no pre-and post-changes within the same region).

None of the participants trained traditional BBP in the intervention, but all participants used BBP in their weekly resistance training (relative 6RM strength of 0.97 in the pre-test). However, the 6RM strength improvement in BBP was ~12–17% whereas the improvement in trained exercise was ~23–39%. Despite the difference in 6RM improvement, the two barbell training groups (Smith machine and Swiss ball) demonstrated no differences in kinematics between trained exercise and the BBP in any of the three lifting regions in the pre- or post-tests. In comparison, a previous study has demonstrated lower 1RM lifting velocity in BBP with increasing relatively strength [11]. It could therefore be speculated that the participants did not lift their “true” 6RM in the pre-test despite two familiarization sessions before. After 10-weeks of training, they were more comfortable of lifting heavier and/or use longer time in the final repetitions (i.e. close to fatigue). Comparing the total lifting time pre- and post-testing support our speculation as all training groups demonstrated twice as long total lifting time in the post-test (S1 Table). For example, has previous studies demonstrated a mean lifting velocity of 0.10–0.17 m/s and examining 1RM in bench press using either powerlifters [35] or strength training men [11]. Mean lifting velocity may therefore be an important measurement in bench press when assessing sets to fatigue. Still, the lifting velocity was stable (i.e. 0.02 m/s) after a 6-week intervention despite a 9.3% 1RM improvement in bench press [11]. Furthermore, the changes observed in the trained exercises (lower barbell velocity, longer time to reach the lifting regions and no difference in vertical displacement) were observed in the sticking and post-sticking region. With similar changes in kinematics between trained chest press exercise and BBP, no training specificity regarding chest-press exercises was observed. Training specificity has previously been demonstrated between contractions forms, movement pattern, joint position and contraction speed [22, 36–38]. The lack of demonstrating training specificity is probably because the exercises are closely related in terms of the muscle groups involved, muscle contraction and lifting pattern [37–40].

For the dumbbell group, differences were observed at both pre- and post-test (Table 3). For the pre-test, the differences were most likely a result of less familiarization lifting 6RM loads in dumbbell chest-press. Typically, the dumbbell chest-press was trained with a higher number of repetitions than tested in the participants`weekly training before participating in the study. However, after performing 10 weeks of 6RM training with dumbbells, the participants may be more familiar of heavy dumbbell chest-press This may, despite the obvious differences between the two exercises (two independent dumbbells vs a barbell) [2, 10], have resulted in the similar post-test changes between the exercises.

There are some limitations which needs to be addressed. Firstly, the present study only examined resistance-trained men. The findings can therefore not be generalized to other populations. Furthermore, the participants trained only one of the three different chest-press exercises twice per week. The acute and chronic effects of these exercises differ [3, 10, 22] and could potentially affect the outcomes (i.e. kinematics in bench press). Furthermore, a greater training volume peer week may result in a different outcome. Importantly, none of the groups trained the BBP, but all trained the exercises weekly pre-intervention, making the BBP a neutral exercise. In addition, only the trained chest-press exercise was included in the warm-up procedures. Including mobilization exercises, general warm-up and more specific warm-up of the shoulders, elbows, wrists and trunk might resulted in better training sessions and performance. Lastly, there is always an inherent risk of cross-talk between neighboring muscles using EMG measurements [41] or not placing the electrodes in identical locations at post-test [26]. Still, anatomical landmarks were used, following previous recommendations and procedures in addition to having the same investigator perform all electrode placements and normalizing the data.

In conclusion, ten weeks with chest-press training resulted in improved strength, decreased lifting velocity and increased the time in each lifting phase in both trained exercise and the none-trained barbell bench press. Similar changes in the sticking region were observed in the none-trained barbell bench press with no differences between the exercises. This demonstrates that lifting kinematics is not specific to a trained chest-press exercise, but improved strength changes the sticking region among resistance-trained men. The authors recommend lifting velocity in chest-press exercises as an important measurement when examining repetition maximum lifts.

## Supporting information

S1 Table Kinematic in trained exercise. (DOCX)
