# Supplementary material for: The effects of ten weeks resistance training on sticking region in chest-press exercises
Source: PLoS One. 2020 Jul 9;15(7):e0235555. doi: 10.1371/journal.pone.0235555 (PMC7347144; doi:10.1371/journal.pone.0235555)
Supplement: S1 Table — (DOCX) [file pone.0235555.s001.docx]

**Supporting table 1: The kinematics pre-and post-training in trained exercise and barbell bench press for the Smith machine group**

| **Lifting phase** | **Exercise** |  | **Vertical displacement (cm)** | **Time (sec)** | **Velocity (m/s)** |
| --- | --- | --- | --- | --- | --- |
| Pre- sticking region | SM# | Pre | 6.07 ± 2.31 | 0.17 ± 0.05 | 0.38 ± 0.10 |
|  | SM | Post | 5.73 ± 3.19 | 0.22 ± 0.18 | 0.31 ± 0.11 |
|  | SW# | Pre | 11.08 ± 8.61 | 0.29 ± 0.18 | 0.42 ± 0.15 |
|  | SW | Post | 5.93 ± 1.31 | 0.21 ± 0.03 | 0.28 ± 0.08* |
|  | DB# | Pre | 16.91 ± 11.15 | 0.33 ± 0.17 | 0.48 ± 0.18 |
|  | DB | Post | 10.22 ± 6.54 | 0.23 ± 0.12 | 0.30 ± 0.13* |
|  | | | | | |
| Sticking region | SM | Pre | 20.26 ± 5.49 | 0.75 ± 0.28 | 0.19 ± 0.08 |
|  | SM | Post | 18.79 ± 7.14 | 1.16 ± 0.54* | 0.08 ± 0.10* |
|  | SW | Pre | 20.14 ± 8.57 | 0.64 ± 0.19 | 0.18 ± 0.21 |
|  | SW | Post | 15.44 ± 6.95 | 1.20 ± 0.72 | 0.01 ± 0.07* |
|  | DB | Pre | 32.84 ± 11.21 | 0.80 ± 0.38 | 0.36 ± 0.15 |
|  | DB | Post | 28.32 ± 12.08 | 1.34 ± 0.57* | 0.05 ± 0.07* |
|  | | | | | |
| Post- sticking region | SM | Pre | 36.78 ± 5.45 | 1.41 ± 0.43 | 0.38 ± 0.08 |
|  | SM | Post | 33.15 ± 10.52 | 2.56 ± 0.90* | 0.29 ± 0.04* |
|  | SW | Pre | 30.44 ± 7.49 | 1.03 ± 0.23 | 0.37 ± 0.11 |
|  | SW | Post | 27.12 ± 7.94 | 2.26 ± 0.74* | 0.23 ± 0.08* |
|  | DB | Pre | 40.12 ± 11.41 | 1.25 ± 0.72 | 0.43 ± 0.14 |
|  | DB | Post | 41.66 ± 6.34 | 2.59 ± 1.19* | 0.23 ± 0.09* |

*significant differences between pre- and post training (p > 0.05). # SM = Smith machine group, SW = Swiss ball group and DB = Dumbbell group
